# Transforming gastrointestinal helminth parasite identification in vertebrate hosts with metabarcoding: a systematic review

**DOI:** 10.1186/s13071-024-06388-1

**Published:** 2024-07-19

**Authors:** Madison L. Miller, Christopher Rota, Amy Welsh

**Affiliations:** Division of Forestry and Natural Resources, West Virginia University, Morgantown, WV USA

**Keywords:** DNA metabarcoding, Helminth parasite, Community diversity, Parasite identification

## Abstract

**Background:**

Gastrointestinal helminths are a very widespread group of intestinal parasites that can cause major health issues in their hosts, including severe illness or death. Traditional methods of helminth parasite identification using microscopy are time-consuming and poor in terms of taxonomic resolution, and require skilled observers. DNA metabarcoding has emerged as a powerful alternative for assessing community composition in a variety of sample types over the last few decades. While metabarcoding approaches have been reviewed for use in other research areas, the use of metabarcoding for parasites has only recently become widespread. As such, there is a need to synthesize parasite metabarcoding methodology and highlight the considerations to be taken into account when developing a protocol.

**Methods:**

We reviewed published literature that utilized DNA metabarcoding to identify gastrointestinal helminth parasites in vertebrate hosts. We extracted information from 62 peer-reviewed papers published between 2014 and 2023 and created a stepwise guide to the metabarcoding process.

**Results:**

We found that studies in our review varied in technique and methodology, such as the sample type utilized, genetic marker regions targeted and bioinformatic databases used. The main limitations of metabarcoding are that parasite abundance data may not be reliably attained from sequence read numbers, metabarcoding data may not be representative of the species present in the host and the cost and bioinformatic expertise required to utilize this method may be prohibitive to some groups.

**Conclusions:**

Overall, using metabarcoding to assess gastrointestinal parasite communities is preferable to traditional methods, yielding higher taxonomic resolution, higher throughput and increased versatility due to its utility in any geographical location, with a variety of sample types, and with virtually any vertebrate host species. Additionally, metabarcoding has the potential for exciting new discoveries regarding host and parasite evolution.

**Graphical Abstract:**

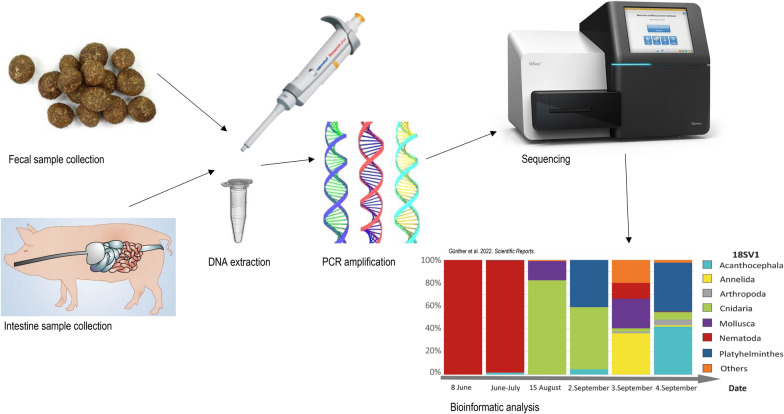

**Supplementary Information:**

The online version contains supplementary material available at 10.1186/s13071-024-06388-1.

## Background

Globally, gastrointestinal parasites are a health concern for many animals including humans. In humans, gastrointestinal parasite infections are common and while most are harmless, some can cause severe illness or death. It is estimated that over one billion humans are currently infected with one or more species of helminth parasites in developing regions of Africa, Asia and the Americas [1]. Gastrointestinal parasites are also common in fish, wildlife, domestic pets and livestock. In goats, an important livestock species in many parts of the world, gastrointestinal parasitism, specifically by nematodes, can result in clinical disease and significant productivity loss [2]. Also, in wild animals, gastrointestinal parasites can have widespread effects, such as reducing individual body condition [3], causing population declines [4] and altering behavior [5]. In wild mandrills, for example, animals will cease grooming activities and avoid parasitized fecal material if they sense an intestinal parasitic infection in a group member [6]. Although gastrointestinal parasites frequently induce negative effects in their hosts, they may also be vital in structuring ecosystems and limiting the abundance of highly competitive species to allow for greater biodiversity [7]. Gastrointestinal parasites may even have uses in treating human health conditions. For example, infection by some nematode species has shown potential in treating human inflammatory conditions such as Crohn’s disease, asthma and multiple sclerosis [8]. Regardless of whether gastrointestinal parasites have a negative or positive impact on their host, the ability to assign accurate taxonomic identification is necessary for diagnosing disease and conducting research.

Gastrointestinal parasites comprise several taxonomic groups, including protozoans, protists and helminths. Helminths, a polyphyletic group of worm-like invertebrate parasites, are deemed one of the most common types of animal gastrointestinal infections [9]. Three phyla belong to the helminth group, namely Nematoda (roundworms), Acanthocephala (thorny-headed worms) and Platyhelminthes, which includes the classes Cestoda and Trematoda (tapeworms and flukes, respectively) [10].

 Historically, light microscopy was the only option available to identify helminths, with identification relying on anatomical and morphological features, such as body length, head shape, number of annuli, sexual organs and shape of stoma [11]. While light microscopy is still a vital method today for describing new species and measuring parasite abundance, using visual identification methods to characterize entire helminth parasite communities are time consuming, require trained taxonomists and can be challenging since morphological characteristics are highly variable among individuals. Moreover, some species of helminths may exhibit identical morphology and be nearly impossible to distinguish using visual identification methods yet are taxonomically classified as unique species with varying ecological niches and host impacts. Therefore, identifying helminth individuals to lower taxonomic groupings can be very difficult using light microscopy [12]. Due to these challenges, newer methods utilizing molecular biology have been established. These techniques range from studying proteins and enzymes to analyzing DNA sequences [12].

DNA barcoding, the precursor to metabarcoding, was proposed two decades ago by Hebert et al. [13] who used short, standardized gene regions to rapidly provide accurate identification of a species. At that time, DNA barcoding was conducted using conventional PCR to amplify the cytochrome* c* oxidase I (COX1 or COI) region followed by Sanger sequencing [13]. In conjunction with morphological identification, DNA barcoding quickly became a useful tool to assess biodiversity, document new species, resolve taxonomic disagreement and help achieve conservation goals [14]. However, this method is limited in that Sanger sequencing is a low-throughput method and can only read the one dominant DNA sequence in each sample [15]. DNA metabarcoding was developed next and is defined as the simultaneous tagging, sequencing and identification of multiple species within the same sample. [16]. Initially, metabarcoding was utilized in microbiology and environmental DNA analyses, but it has since also become a staple methodology for diet assessments as it is accurate, allows lower taxonomic resolution and can be used with little prior knowledge of diet composition [17, 18]. The ability to identify species without prior knowledge of community composition is a strong advantage of using metabarcoding instead of DNA barcoding methods, since DNA barcoding requires a priori knowledge of the species expected to be found in samples in order to use the correct primers. The benefits of metabarcoding have been noted by researchers in other fields, including parasitologists. Consequently, metabarcoding has increased significantly in popularity for application in helminth parasite identification in the last decade.

Aivelo and Medlar [19] reviewed four helminth metabarcoding papers with the purpose of examining the usefulness of this method in parasitological research and assessing its benefits and limitations. These authors reported that metabarcoding provided fast and extensive parasite community composition analysis while allowing for non-invasive sampling methods. In the time since their review was published, the number of scientific publications using metabarcoding to identify helminth parasite communities has continued to steadily increase. In these studies, methodological approaches vary greatly. As such, there is a strong need to synthesize and compare techniques used across recent gastrointestinal helminth parasite metabarcoding studies.

In this article, we report our thorough review of peer-reviewed literature that used metabarcoding to identify gastrointestinal helminth parasites in vertebrate hosts. We use this literature review to provide an overview of the metabarcoding process steps from sample collection to bioinformatic analysis and include considerations for new users interested in developing their own study. We also examine the benefits and limitations of helminth parasite metabarcoding and discuss possible future directions for the field.

## Methods

To synthesize gastrointestinal helminth parasite metabarcoding approaches, we conducted a systematic literature survey using Google Scholar, Web of Science and PubMed from February 2023 to August 2023 for relevant articles using the key words “metabarcoding,” “nematode,” “cestode,” “trematode,” “parasite,” “fecal,” “helminth,” “intestine,” “platyhelminth,” “acanthocephalan,” “amplicon sequencing,” “molecular barcoding,” “flatworm,” “threadworm,” “thorny-headed worm” and “stool” in various combinations of one to three keywords. The resultant papers were screened for the following criteria: (i) must be peer-reviewed, original research; (ii) must have used metabarcoding to detect helminth parasites from fecal matter, the cecum or intestines of host; and (iii) host species must be a vertebrate. We excluded studies that used metabarcoding with the main goal of detecting diet items and only detected parasites incidentally, as these studies may not have used methods to ensure detection of full parasite communities. We also excluded studies utilizing environmental samples such as water or soil, or samples with a high risk of environmental contamination such as latrines, middens and cesspits.

After filtering the initial literature search results, we conducted full-text reviews for retained studies. For each study, we extracted the year of publication, host species, host taxonomic group, helminth parasites detected, type of sample used, DNA extraction method used, genetic marker used, sequencing platform, bioinformatic pipeline and database used, whether parasites were isolated from samples before DNA extraction and location of sampling sites. If necessary, appendices and supplementary materials were downloaded and examined to locate information. We also scanned literature for sample site locations and recorded latitude/longitude if this information was supplied in the text; if not supplied, we used Google Maps to find the nearest latitude/longitude point of the sampling site. If specific sampling locations were not mentioned in the article, we created a latitude/longitude point in the center of the state/province/country mentioned, and then imported latitudes and longitudes into ArcGIS Pro version 3.0.3 (ESRI, Redlands, CA, USA) to create a map of sampling locations for studies included in our review.

**Fig. 1:**
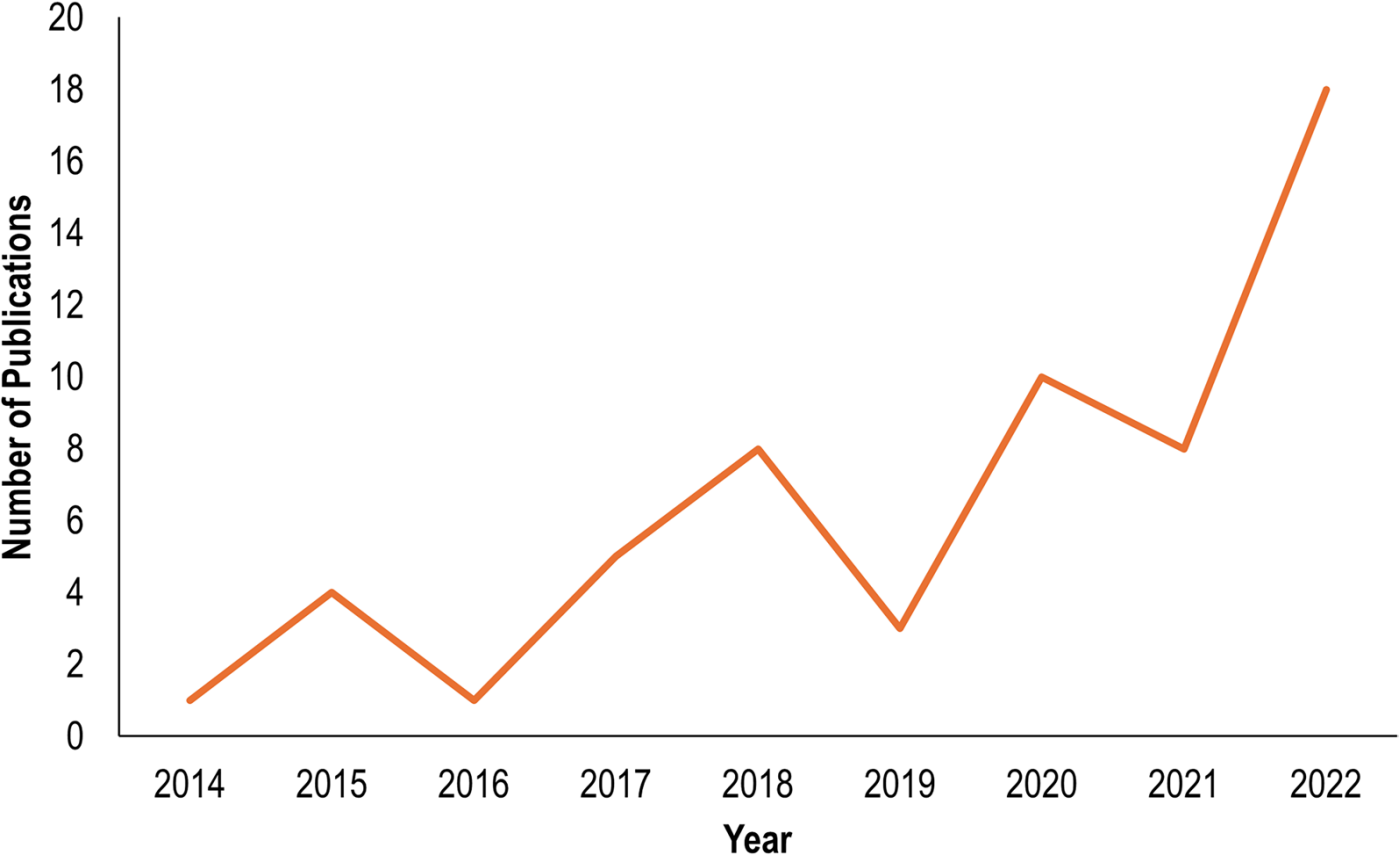
Number of studies using metabarcoding to assess gastrointestinal helminth parasites in vertebrate hosts recovered from a systematic literature search conducted during the period February 2023 to August 2023. Four studies were published in 2023 (not shown) by the conclusion of the data collection period

**Fig. 2:**
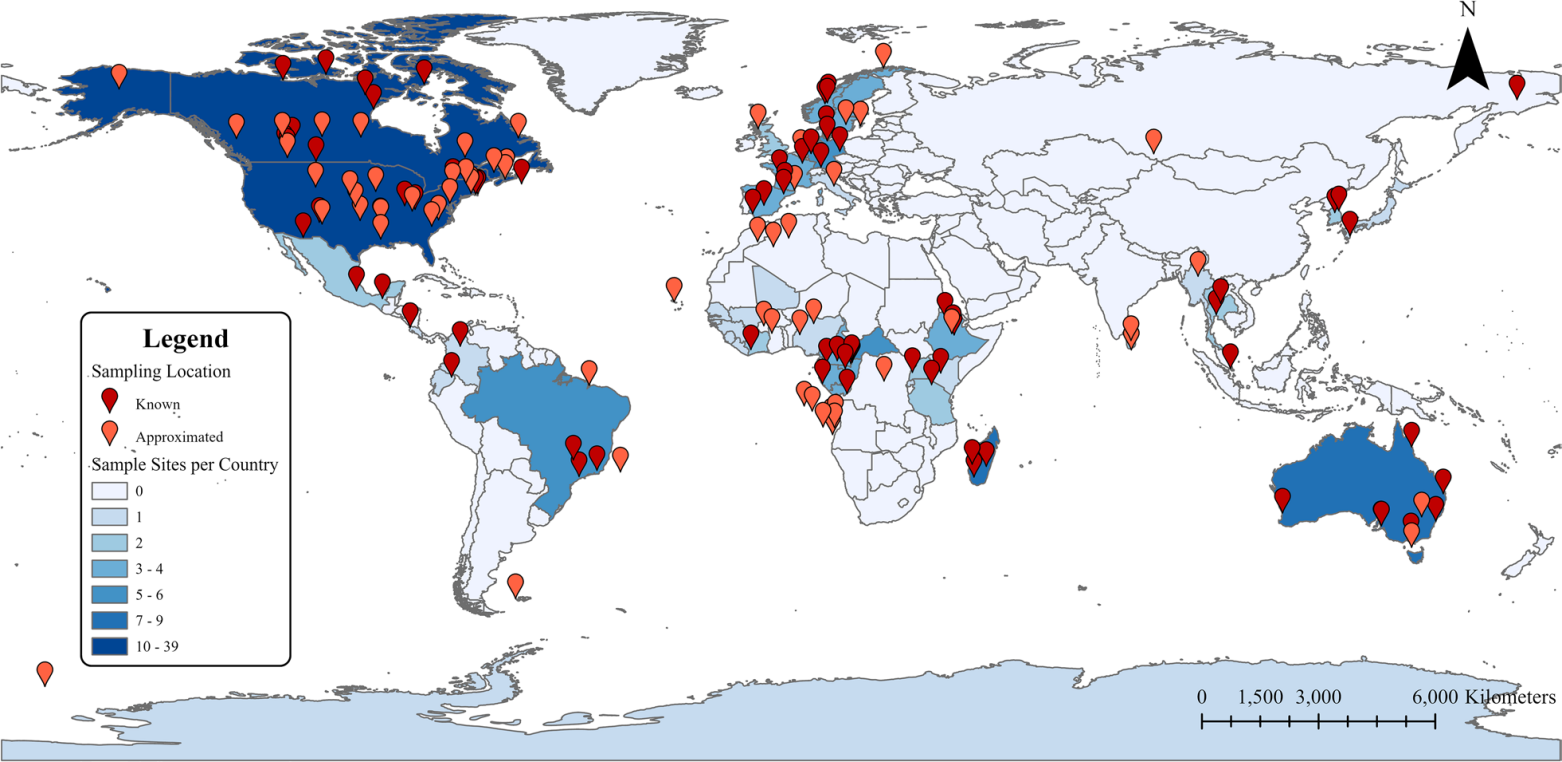
Geographic distribution of sampling locations in 62 helminth metabarcoding studies published from 2014 to 2023. The pins represent the locations of sample sites utilized in studies, with the dark-red pins showing exact locations and the light-red pins showing approximated latitude/longitude coordinates. Blue shading (from light to dark) represents the number of studies conducted per country (see scale at bottom left of figure). The map was created with ArcGIS Pro version 3.0.3 (ESRI, Redlands, CA, USA)

Our literature search yielded 62 articles that spanned the time period from 2014 to 2023 (Additional file 1: Table 1). The number of studies published per year increased steadily over this time period, with 29.0% of studies included in our review published in 2022 (Fig. 1). Studies were conducted in 43 different countries, with 23.8% of study sites located in Canada, likely due to the Nemabiome method developed in this country by the Gilleard laboratory (https://www.nemabiome.ca/) (Fig. 2). Each step of the metabarcoding process was examined, and specific techniques were identified in the studies from our literature review. It is worth noting that some studies used multiple techniques for one step, which may result in the sum of numerators in ratios > 62.

## Steps in the metabarcoding process

### Sample collection

Parasitic gastrointestinal helminths can be identified with DNA metabarcoding using several different host sample types. While helminth life-cycles differ among taxonomic groups, species will spend a portion of their lifetime located in the gastrointestinal tract of their host [20–23]. Eggs from reproducing adults may also be expelled during host defecation [24]. In our review of 62 papers, samples originated from three sources: fecal matter (88.7%), gastrointestinal tract (12.9%) and cloacal swabs (1.6%). However, there are several considerations to take when choosing a sample type for a helminth parasite metabarcoding study (Table 1).

**Table 1:** Considerations for new users developing a helminth metabarcoding protocol

Step	Method	Pros^a^	Cons^a^
1. Collect samples	Fecal material	Non-invasive	Risk of environmental contamination
		Low effort to collect	Parasite species that are actively reproducing may make up larger portion of sequence reads
		Can obtain a large sample size	DNA may be degraded and/or PCR inhibitors may be present in fecal matter
	Gastrointestinal tract tissue	Can detect parasites that are not actively reproducing	Invasive
		Environmental contamination is limited	Obtaining a large sample size may be difficult, especially for imperiled species
	Cloacal swab	Non-invasive	Only available for certain host species
		Low effort to collect	Swab may miss parasite species located higher up in orifice
2. Extract DNA	Traditional technique (i.e. isopropanol precipitation, phenol–chloroform)	May already have reagents in laboratory, low cost	May need to troubleshoot to validate protocol
		High DNA yield	Lower detection rate of species present in sample
	Commercial kit	DNA kits made specifically for fecal matter to decrease PCR inhibitors	Higher cost
		High detection rate of species present in sample	Variable DNA yield depending on sample type and kit used
	Isolating parasites from sample before extraction	Limits PCR inhibitors in fecal matter	May lose some adult parasites if utilizing a sieving method focused on collected eggs, avoidable if using co-proculture and extracting whole sample
			Time-consuming
	Lysis procedures (i.e. bead beating)	Increase DNA yield of species with hard eggshells	Time-consuming, additional costs
3. Amplified marker region	Nuclear ITS	Good for species identification due to highly variable sequences between species	ITS region may not as suitable for inferring phylogenetic relationships due to nucleotide substitution saturation
	Nuclear rRNA (28S, 18S)	Highly conserved, good for determining phylogenetic relationships	Lower resolution of species-level identification
		Easier primer design due to more universal primers and reference sequences available	Only a small proportion of sequence reads are specific to nematodes for 18S
	Mitochondrial COX1	Universal primers available	High sequence variation in COX1 gene in helminths compared to other groups leads to low PCR amplification success and limited taxa identification
	Mitochondrial 16S	Good phylogenetic and species resolution	Used less frequently in studies causing fewer reference sequences available
	Mitochondrial 12S	Good phylogenetic and species resolution, may recover more platyhelminth and nematode species than 16S	Used less frequently in studies causing fewer reference sequences available
4. Sequencing platforms	Illumina (Illumina Inc., San Diego, CA, USA)	Low error rates	Maximum read length is appprox. 500 bp (for paired end sequencing)
		Produces short reads, which can provide high read numbers per sample	Short reads may have lower accuracy in assigning taxonomy
		Available at most genomic core facilities, well-established procedures	Less power in determining phylogenetic relationships
	Pacific Biosciences of California, Inc. (PacBio; Menlo Park, CA, USA)	Produces long reads, up to 10,000 bp long	Primer pairs need to be developed to cover the longer fragments, lower read depth
		Long reads may be necessary if using long marker regions or a combination of marker regions	Less available at genomic core facilities than Illumina platforms
		Long reads are better for phylogenetic inferences	Higher error rate than the Illumina method
5. Bioinformatic analysis and database	NCBI GenBank	Database is largest, contains sequences from all phyla	Not all sequences are annotated
		Compatible with any genetic marker	Mislabeled sequences may be present, database is less curated than more custom databases
	Nemabiome ITS2 database	Clear protocols developed	Only contains sequences from phylum Nematoda
		Database is updated frequently	Only compatible with ITS2 genetic marker
	SILVA rRNA database	Quality checked and regular updates	Only compatible with rRNA markers (18S, 16S, 28S, 23S)
	Protist Ribosomal Reference database (PR^2^)	Annotated, allows for interpretation of the structure and function of specific genes	Only compatible with 18S rRNA and limited 16S genetic markers

*COX1* Cytochrome *c* oxidase 1,* ITS *internal transcribed spacer,* NCBI* National Center for Biotechnology Information, *rRNA *ribosomal RNA

^a^Pros and cons are listed for each step of the process

As a sample type, fecal matter has many advantages when used for gastrointestinal parasite metabarcoding because (i) its collection does not depend on trapping animals or lethal sampling; (ii) it is easy to obtain a large sample size; (iii) it can be collected from elusive or rare species; and (iv) it is generally a low-cost endeavor to collect [19, 25, 26]. However, fecal matter may contain PCR inhibitors and the DNA in fecal matter may be fragmented or degraded, causing potential issues in downstream molecular analysis [27]. While sloughed cells, free DNA and deceased parasites are released into feces and therefore may be detected during metabarcoding, helminth parasite taxa have different reproductive rates, and the more fecund species may therefore be overrepresented in fecal samples [28, 29]. Also, fecal matter is susceptible to contamination by helminths in the environment after it is deposited. Limiting external helminth parasites may be accomplished by using only the interior of the feces sample and avoiding the outer layers [26], by collecting fecal matter directly from the rectum or by collecting fecal matter immediately after defecation while observing the hosts [30].

Alternatively, using gastrointestinal tract samples for helminth parasite metabarcoding largely eliminates the risk of environmental contamination. An additional benefit of using gastrointestinal tract samples is that a more accurate representation of the helminth community is obtained since the entire gastrointestinal tract will be assessed. However, using samples from gastrointestinal tracts requires the host to be deceased, which may not always be feasible, especially with threatened or endangered host species. Using swabbing methods (cloacal, fecal, rectum) may limit PCR inhibitors in fecal matter, but could lead to missing parasites located higher up in the gastrointestinal tract.

Sample storage is another important consideration as preservation methods can impact the quality of DNA [31]. If possible, extracting DNA from the samples immediately is advised to prevent degradation from high temperatures [32, 33]. Alternatively, fecal samples can be stored in 70% ethanol at − 18 °C, which will provide suitable conditions for both DNA extraction and morphological analysis of parasites in the sample using a method such as fecal flotation [19]. If ethanol is used to store fecal samples, it is important to add a sufficient volume relative to the sample quantity to prevent degradation and to completely remove ethanol from samples before DNA extraction to avoid negatively impacting downstream analyses. Other potential fecal preservation methods include preservation buffers such as RNAlater (Ambion, Thermo Fisher Scientific, Waltham, MA, USA) or DNA/RNA Shield (Zymo Research, Irvine, CA, USA), although the effectiveness of these solutions has not yet been tested in helminth metabarcoding studies. It is recommended that gastrointestinal tract samples be stored in 70–90% ethanol at − 20 °C [34, 35] or at − 80 °C until extraction [36, 37].

### DNA extraction

Selection of a DNA extraction method is an important step to ensure that high-quality DNA is isolated from the collected samples at concentrations sufficient for downstream genetic analyses. In our literature review, the majority of researchers (72.6%) used a commercial extraction kit to isolate DNA from their samples. The next most common DNA extraction method among the papers in our review (9.7%) was a method following the internal transcribed spacer-2 (ITS-2) ribosomal DNA (rDNA) nemabiome metabarcoding protocol [38]. The remaining studies used traditional extraction processes, such as isopropanol precipitation or phenol–chloroform, or DNA extraction methods that were not specified.

Several considerations need to be taken into account when choosing a DNA extraction protocol for a helminth metabarcoding study (Table 1). For example, an additional step that 46.8% of researchers included in our review took during the extraction process was isolating the parasites from the sample before extracting DNA. Several methods are possible to isolate helminth parasites, such as the Baermann method, flotation methods, larval cultures for fecal samples or manual removal of adult helminths from the digestive system [39]. Isolating helminth parasites from the sample for DNA extraction can significantly improve the sensitivity of molecular analyses by removing inhibitors and increasing concentration of parasites [40]. However, parasite removal may also increase the risk of losing individual specimens if using sieving methods and increases laboratory time.

One benefit of using commercial kits for DNA extraction is that they are designed to mitigate the effect of PCR inhibitory compounds in the starting materials, which is a concern when using fecal matter [41]. While traditional DNA extraction methods using laboratory reagents such as phenol–chloroform have been found to yield higher starting concentrations of DNA, commercial kits are associated with higher detection rates of species in the sample after PCR [42]. Modifications to laboratory protocols may be necessary to maximize helminth DNA yield. For example, it may be difficult to disrupt the eggshell of some helminth species, including many nematodes [19, 40, 43]. If this issue is encountered, bead beating during the lysis stage has been shown to increase DNA yield as it helps break down the outer layer of eggs of nematode species [40, 44]. This example highlights the importance of using positive controls during sample processing to determine whether parasites present in a host are being detected.

Positive controls are a vital part of the metabarcoding process to ensure that the chosen methodology is able to detect the species present in collected samples. It is beneficial to include mock communities, which are simulated communities of known and pre-defined species composition, in the metabarcoding process to test the reliability of the parasite detection process [45]. It is also possible to calculate correction factors from mock communities with exact known proportions of parasite species to reduce sequence representation biases from the unknown samples [38, 46]. Negative controls are also an essential addition for quality control and can be implemented during extraction by performing a DNA extraction on a water sample simultaneously with the unknown samples. Negative controls should also be included in all downstream analyses to monitor contamination, and separate negative controls can be included at each stage to help determine the source of contamination if it occurs [47].

### Amplified marker region

A key step in the metabarcoding process is identifying a marker region at which to amplify DNA [33]. Genetic markers are segments of DNA that can provide molecular information enabling the differentiation of taxa [48]. There are several marker regions from which to choose for gastrointestinal parasite metabarcoding, within both mitochondrial DNA and nuclear DNA. Also, multiple genetic markers can be targeted simultaneously if the aim is to increase detection coverage. In our literature review, the majority of studies utilized nuclear markers, with 46.8% using the 18S ribosomal RNA (rRNA) region and 43.5% using the ITS-2 region. Of studies that used the mitochondrial region, COX1 was the most popular marker (9.7%). Other less used nuclear regions included 28S rRNA and other mitochondrial regions (e.g. 12S rRNA and 16S rRNA).

**Fig. 3:**
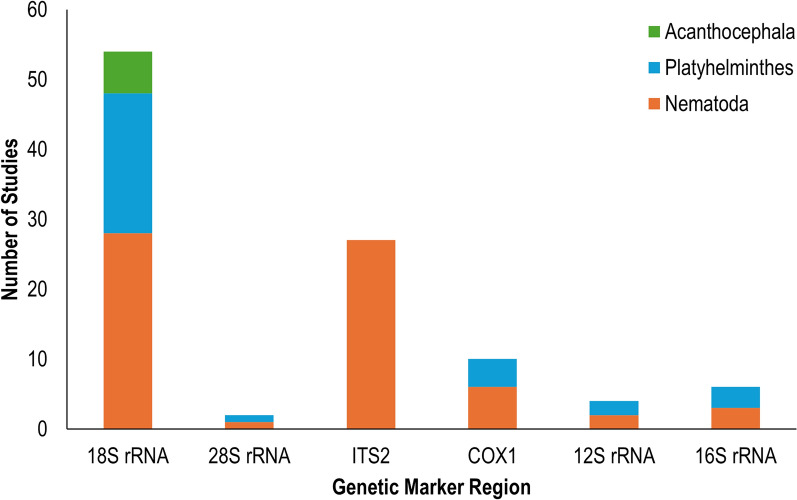
Phylum detection across the 62 studies included in our review of gastrointestinal helminth parasite studies associated with the gene markers 18S ribosomal RNA (*18S rRNA*), 28S ribosomal RNA (*28S rRNA*), nuclear internal transcribed spacer 2 (*ITS2*), cytochrome* c* oxidase I (*COX1*), 12S ribosomal RNA (*12S rRNA*) and 16S ribosomal RNA (*16S rRNA*)

The differences in usefulness and resolution among genetic markers is highly related to the degree of sequence variation within the marker [48]. Mitochondrial DNA evolves faster than nuclear DNA, producing a higher degree of sequence variation which may lend itself to being a useful marker for resolving lower taxonomic levels. Conversely, nuclear DNA and particularly the nuclear rRNA genes, are more conserved and are a potentially helpful source to resolve higher taxonomic levels. A recent study compared the utility of four classes of genetic markers for helminth species identification, including nuclear ribosomal internal transcribed spacers (ITS1 and ITS2), nuclear rRNA (18S and 28S), mitochondrial rRNA (12S and 16S) and mitochondrial protein-coding genes (COX1 (COI and COII), ctyb and NAD1) [48]. The authors found that nuclear and mitochondrial rRNA markers were best for helminth molecular systematics while mitochondrial protein-coding and rRNA genes were suitable for molecular identification. Other factors to consider when choosing a genetic marker region include the availability of reference sequences in databases. Mitochondrial markers such as 16S and 12S are less frequently used in helminth metabarcoding studies and, therefore, fewer reference sequences are available in databases compared to popular genetic markers such as 18S and ITS [49]. Additionally, universal eukaryotic primers are available for frequently used genetic markers in the rRNA region, including 18S and 28S, which can make designing a study easier. In our review, many studies were only interested in describing Clade V nematode parasites and utilized a sequencing pipeline with the ITS2 marker established by Avramenko et al. [38] [30, 50–54]. Studies in our review that were interested in identifying as many helminths as possible across all phyla used the 18S marker to successfully identify species from Nematoda, Platyhelminthes (both Cestode and Trematoda classes) and Acanthocephala [55–57] (Fig. 3).

**Fig. 4:**
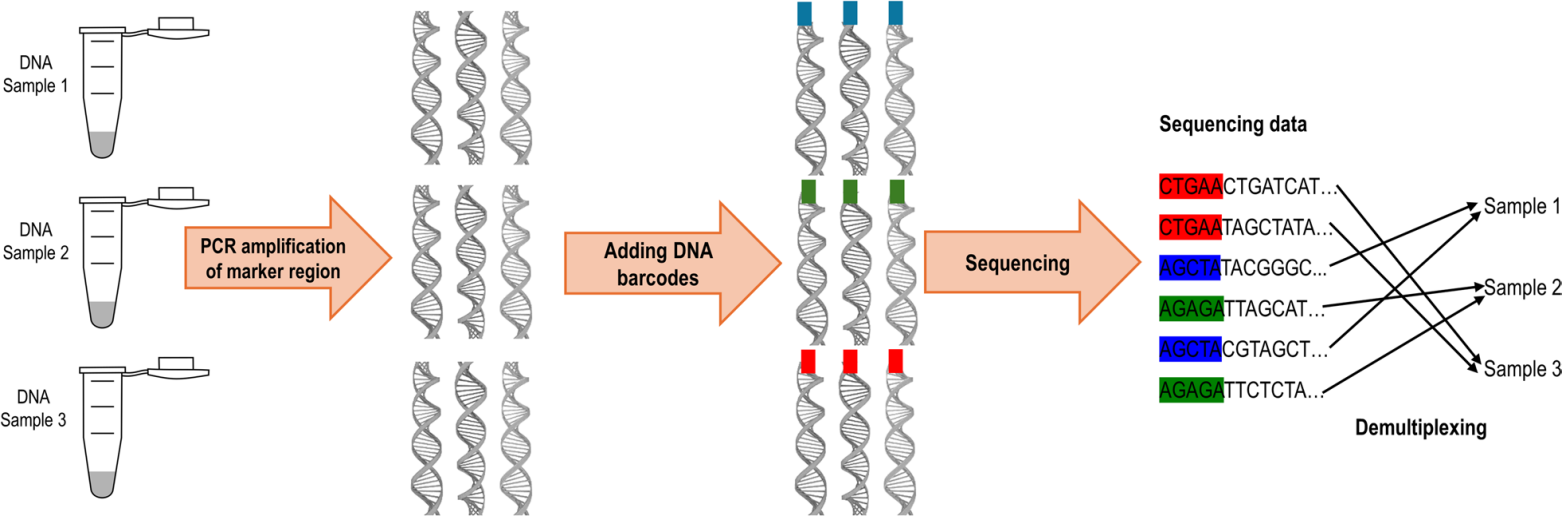
Schematic overview of the steps in a PCR multiplex protocol for three samples

Once a gene marker region has been chosen, PCR primers need to be selected that will amplify DNA within the chosen gene marker from individual helminths. The studies included in our literature review used a wide variety of primer sequences, ranging from custom designed primers to universal eukaryotic primers. As such, we did not consolidate primer sequences across our review studies due to the large amount of variation across studies. While there are no universal primers across all taxa, primers should be chosen that are conservative enough to amplify DNA from taxonomically close organisms while containing variable sites across species, allowing for taxonomic assignment [31, 58, 59]. Universal primers such as the universal eukaryotic primer set (Euk1391f and EukBr) were a common primer choice for studies in our review [36, 56, 60–62]. However, universal primers such as these may also co-amplify non-target sequences, such as host DNA, diet DNA or fungal DNA, thereby consuming sequencing efforts. Blocking primers can be included during PCR to limit the amplification of non-target sequences. For example, a mammalian host blocking primer preferentially binds to mammal DNA but is modified with a C3 spacer on the 3’ end to inhibit elongation [63]. Also, universal primers do not perfectly match the DNA of all species, and there may be a template-primer mismatch during PCR [64]. This could result in some species amplifying at higher rates than others and, therefore, the final proportion of sequences may not reflect the true proportion of parasite species. Including a mock community with known proportions of parasite species can be useful to calibrate for PCR amplification bias [33]. A primer database is available in the Barcode of Life Database (BOLD) system where users can search from a list of published primers [65]. An additional helpful resource for metabarcoding primer selection is the PrimerTC tool which uses global pairwise alignment to assess the percentage of similarity between a chosen primer sequence and a reference database [66]. Amplification of extracted DNA with the chosen primers is then performed to prepare a genomic library. This occurs as a PCR with sample-specific nucleotide identifiers (i.e. barcodes) assigned to amplicons in each sample. Samples can then be pooled into a library before sequencing, and barcodes allow for sequences to be assigned back to their original sample (Fig. 4) [67].

### Sequencing platforms

In the next step, the prepared genomic libraries will need to be sequenced, a process that determines the order of nucleotides, or bases, in a nucleic acid molecule. This can be completed on several different sequencing platforms using next-generation sequencing technology, which is a high-throughput method that allows for rapid sequencing of millions of DNA molecules simultaneously [68]. In our literature review, Illumina sequencing platforms (Illumina Inc., San Diego, CA, USA) were used in most studies (85.5%). The Illumina MiSeq is currently the most popular platform for DNA metabarcoding studies because it provides reasonable read depth and has low error rates and well-established bioinformatic procedures at an affordable cost [31]. Other sequencing options used in the reviewed studies include the Roche 454 (8.1%; although this technology has been discontinued) and PacBio sequencers (4.8%; Pacific Biosciences of California, Inc. [PacBio], Menlo Park, CA). One consideration when choosing a sequencing platform is the price. The per-cell costs of common sequencing platforms used in our studies are relatively similar, with the Illumina MiSeq v3 300 bp at $2250 USD and the PacBio Sequel IIe at $2225 USD (NC State University; https://research.ncsu.edu/gsl/pricing/). Another consideration when choosing a sequencing platform is the desired sequencing depth, i.e. the number of times that a given nucleotide in the genome has been read in a reaction (Table 1). Producing long reads may be necessary when using long markers such as 18S or a combination of 12S and 16S as a single marker [33]. A recent comparison of sequencing platforms showed that while the long reads produced by PacBio may have a higher accuracy in assigning taxonomy, Illumina short reads provide higher read depth per sample [69]. Optimal read depth may depend on study goals. For example, high read depth may be beneficial for samples that have low species abundances while, alternatively, lower read depths may be sufficient for sequencing platforms with low error rates. There is currently no consensus on an optimal read depth, but an average depth of 55,000–60,000 reads per sample is common in metabarcoding studies [31, 70, 71].

### Bioinformatic analysis and databases

Raw data generated during sequencing will contain millions of reads from DNA amplicons at the genetic marker region chosen. The bioinformatic analysis workflow follows the general steps of demultiplexing samples, merging paired-end reads, quality filtering, operational taxonomic unit (OTU) curation and taxonomic assignment [31]. Taxonomic assignment involves comparing reads generated in the study against a publicly available reference database or study-generated reference sequences [72]. This comparison will allow the sequences produced from metabarcoding to be matched with known sequences and assigned to taxonomic groups. It is important to note that it may not be possible to assign every sequence to a species, and for this reason many studies instead utilize OTUs, which are clusters of sequences that have a sequence identity above a given threshold, frequently 97% [73]. Alternatively, denoising approaches, such as amplicon sequence variants (ASVs), can be used and are based on predicting and correcting actual sequencing errors (noise) before forming clusters [74]. In our literature review, researchers used several bioinformatic databases to identify sequences, including the NCBI GenBank [75] (59.7% of studies), the Nematode ITS2 rDNA database [76, 77] (24.2% of studies), SILVA [78] (19.4% of studies), PR2 [79] (4.8% of studies) and databases made from unspecified publicly available reference sequences (4.8% of studies). An important aspect to consider when deciding which bioinformatic database to use is whether the genetic marker used in your study is compatible with the sequences in the database (Table 1). For example, while researchers included in our review frequently used the Nemabiome ITS2 database, this database only contains sequences from the phylum Nematoda. Similarly, the SILVA database is only compatible with rRNA markers such as 18S, 16S and 28S. The NCBI GenBank is the largest database available, containing 2.9 billion nucleotide sequences for 504,000 formally described species as of January 2023 [80], which allows for the most comprehensive assessment of taxa in a sample. The main tradeoff with using a large database is that sequence mislabeling may occur and not all sequences may be annotated (i.e. having the location and functionality of genes along the sequence described) [81]. Smaller databases, such as SILVA, PR2 and Nemabiome ITS2 rDNA, are quality checked and updated frequently, and most sequences are annotated [76]. It is also possible to create a custom database using highly accurate, well-annotated sequences, or to conduct a sequencing experiment on known positive controls for comparison with unknown samples. Once a bioinformatic database has been selected, bioinformatic software programs are used to process raw sequencing data, perform quality control and generate a pipeline using algorithms to match unknown sequences with taxonomic assignments. Mock communities are also beneficial during the analysis step as they can help test for optimal bioinformatic parameters along the pipeline [33].

Several software programs and pipelines are used to analyze metabarcoding data, such as QIIME, DADA2, Mothur, USEARCH and OBITools. In our review, the majority of researchers used DADA2 [82] (30.6%) followed by Mothur [83](22.6%). In-depth reviews of current bioinformatic software and pipelines available with user guides are provided in Deiner et al. [72], Mathon et al. [84] and Hakimzadeh et al. [85].

## Discussion

In our review of gastrointestinal helminth metabarcoding studies, we found that methodology varied widely. Metabarcoding techniques have been shown to vary in other areas of research, including diet studies [18], marine ecosystem studies [33, 70], entomology studies [31] and environmental DNA studies [72]. Our results found differences in nearly every aspect of the metabarcoding process, including sample type utilized, DNA extraction method used, genetic marker region targeted, sequencing platform used and bioinformatic database choice. Some aspects of the metabarcoding process may be able to vary and achieve similar results. For example, studies have found that several different commercial DNA extraction kits perform similarly in PCR amplification of gastrointestinal helminths from fecal matter samples [42, 86]. However, other steps need careful consideration to ensure that the chosen methodology will yield the desired outcome. Some bioinformatic databases are only compatible with specific genetic marker regions. The SILVA bioinformatic database [78] only contains sequences generated by rRNA markers such as 18S or 16S, and the Nemabiome ITS2 database [76, 77] has only sequences from phylum Nematoda.

We found that a main reason for discrepancies in methodology among studies in our review was differences in the goal of the research. For example, many studies were only interested in assessing gastrointestinal Clade V nematode parasites in the host and therefore utilized a well-established sequencing pipeline using the ITS2 marker created by Avramenko et al. [38] [30, 50–54]. Alternatively, the authors of one study were interested in obtaining a comprehensive assessment of the helminth parasite community in wolverine (*Gulo gulo*) hosts and used both a nuclear and a mitochondrial genetic marker (COX1 and 18S), with both intestinal content and fecal samples, to assess parasites at all locations along the gastrointestinal tract [62]. Another objective that shaped methodology choices in some studies was the ability to assess host diet and host gastrointestinal parasites simultaneously. For example, molecular markers and primers that would amplify both diet items and parasite invertebrate species from host fecal samples were utilized in studies conducted by Cabodevilla et al. [87] and Günther et al. [57].

One area that the helminth metabarcoding field would benefit from is for researchers to perform studies that directly compare various aspects of metabarcoding methodology. While we were able to give advantages and disadvantages for certain techniques based on related research, few studies in our review directly tested the efficacy of different techniques for gastrointestinal helminth metabarcoding and rather used only one technique for each step of the metabarcoding process. For example, it would be beneficial to test the performance of various genetic marker regions in recovering gastrointestinal helminth parasite communities from the same sample types while controlling for all other aspects of the metabarcoding process. This would help clarify the question of whether certain genetic markers are better than others in assessing helminth parasite community diversity. Although metabarcoding is largely considered to be a superior tool in comparison to traditional identification methods, there are a few limitations to consider before developing a helminth parasite metabarcoding protocol.

While using metabarcoding to identify gastrointestinal helminth parasites has many benefits, there are several challenges that remain. One such challenge is the cost and bioinformatic expertise required for metabarcoding in comparison to traditional visual identification techniques, which may be prohibitive to some groups. Sequencing alone can cost upwards of $2000 USD for a single 96-sample plate. However, the price of sequencing technology is rapidly decreasing, which will likely make metabarcoding more accessible to users in the future [33]. Another challenge of metabarcoding is that there is currently no consensus to the degree that metabarcoding is quantitative, or that sequence reads correspond with the abundance of species in a community [19, 31, 88]. Some studies have found a significant positive correlation between sequence read number and parasite abundance [89, 90]. A recent meta-analysis suggests that a weak quantitative relationship may exist between metabarcoding sequences and biomass in a sample [88]. However, there was still a large degree of uncertainty in this result, likely due to variation in techniques used among studies. For this reason, it is advisable to rely on visual identification methods or to calculate correction factors from mock communities for accurate parasite abundance data [38, 46].

Another shortcoming of using metabarcoding to assess community diversity is that it may not always identify all parasite species present in the gastrointestinal tract of the host. This is also a limitation with traditional visual methods, in that species present in fecal matter do not always correlate with the number of adults present in a host [28, 29]. Factors such as reproductive rates, seasonality and time of sample collection can influence the number of parasite eggs found in a fecal sample [91, 92]. For example, some species may have higher reproductive rates and eggs will be overrepresented in fecal matter, resulting in increased amplification of DNA sequences [19]. This problem can be somewhat mitigated by utilizing whole intestinal tracts in sampling at the detriment of a more invasive sample collection. Parasite species may also be undetected during metabarcoding if their sequences are not amplified by the chosen primers or sequences are not present in the database utilized. However, metabarcoding could be used to detect new species by comparing novel sequences generated during sequencing with similar species using phylogenetic trees [93].

The ability of metabarcoding to quickly and comprehensively identify entire communities using genomics creates potential for exciting new discoveries in the parasitological field. One potential area where future research could use metabarcoding is characterizing previously unknown parasite communities from rare or elusive host species. For example, de Vos et al. [94] used metabarcoding with fecal samples opportunistically collected from blue whales (*Balenoptera musculus indica*) and documented the first record of acanthocephalan parasitic worms in this host species in the Northern Indian Ocean. Another novel field that metabarcoding could be used in is the evolutionary associations between helminth parasites and their hosts. Metabarcoding produces large amounts of sequence data for helminth parasites which could be used beyond simple identification and instead used to create phylogenetic trees. These data could lead to interesting insights into helminth evolution in relation to their hosts’ evolutionary history. For example, metabarcoding was used to characterize strongylid nematode community composition in western lowland gorillas (*Gorilla gorilla gorilla*) and agile mangabeys (*Cercocebus agilis*), and it was discovered that Necator formed two distinct clades, one grouping with a species originally described in humans, and the other previously described in humans and lowland gorillas [95].

An important future direction for parasite metabarcoding methodology is to continue incorporating new technological advances, particularly in the sequencing realm. Additional platforms, such as Nanopore DNA sequencing (Oxford Nanopore Technologies, Oxford, UK), have the potential for use in gastrointestinal parasite metabarcoding studies due to the portability of equipment, increased read lengths and low operating costs [96, 97]. For example, a single cell run using the Flongle platform (Oxford Nanopore Technologies) costs $90 USD (https://nanoporetech.com/products/sequence/flongle). Another necessary future direction for gastrointestinal parasite metabarcoding is to continue evaluating the relationship between sequence reads and parasite abundance. Variation in quantitative performance across studies is to be likely related to primer biases, resulting in uneven amplification or mismatches in target DNA sequences [88, 98]. Future research could seek to quantify and adjust for primer biases so sequence reads could accurately predict parasite abundances in a sample.

## Conclusions

Metabarcoding has emerged as an exceedingly useful tool for many areas of biological research in the last few decades. Using metabarcoding to assess gastrointestinal helminth parasite community diversity is preferable to traditional visual methods because it is high-throughput and time-effective, and provides lower taxonomic resolution. This method can be used to assess intestinal parasite communities in a wide range of vertebrate host species in virtually any geographic location across the globe. Gastrointestinal helminth metabarcoding can also be combined with other molecular techniques to simultaneously reveal insights into host population genetics, host microbiome or host diet ecology. New users should make sure to carefully choose each step of the metabarcoding protocol, especially genetic marker region and bioinformatic database choices, to best align with the goal of their study. To our knowledge, this is the most comprehensive literature review of research utilizing metabarcoding techniques to assess gastrointestinal helminth parasite communities in vertebrate hosts to date. While there are still challenges that the metabarcoding field needs to overcome regarding measuring parasite abundance, this method is an overall effective way to assess gastrointestinal helminth parasite communities in vertebrate hosts.

### Supplementary Information

**Additional file 1: Table S1.** Information extracted from the 62 studies included used in our literature review, including citation, year of publication, host species, host taxonomic group, genetic marker used, helminth parasites detected, type of sample used, DNA extraction method used, sequencing approach used, bioinformatic pipeline used, bioinformatic database used and location of sampling sites.

## Data Availability

All data used in this study is available in Additional file: Table 1.

## Abbreviations

ASVs: Amplicon sequence variants
BOLD: Barcode of Life Database
ITS: Internal transcribed spacer region
OTUs: Operational taxonomic units
rRNA: Ribosomal RNA
PCR: Polymerase chain reaction
